# Fondaparinux Failure in Heparin-Induced Thrombocytopenia With Acute Limb Ischemia: A Case Report and Literature Review

**DOI:** 10.7759/cureus.85627

**Published:** 2025-06-09

**Authors:** Adam Bowen, Dania Baraka, Khaleel Quasem, Alfarooq Alshaikhli, Maha Bayya

**Affiliations:** 1 Internal Medicine, Mclaren Greater Lansing Hospital, Lansing, USA; 2 Internal Medicine, McLaren Greater Lansing Hospital, Lansing, USA; 3 Hematology and Oncology, Mclaren Greater Lansing Hospital, Lansing, USA

**Keywords:** acute limb ischemia (ali), anticoagulation, argatroban, direct thrombin inhibitors, fondaparinux failure, heparin-induced thrombocytopenia (hit), hit management, platelet factor 4 (pf4), thrombosis, thrombotic complications

## Abstract

Heparin-induced thrombocytopenia (HIT), a prothrombotic disorder caused by heparin-dependent antibodies, is often treated with fondaparinux, generally yielding positive outcomes. A 69-year-old male with a history of stage IIIb small cell lung cancer developed severe HIT (platelet count nadir, 11 × 10⁹/L) after receiving heparin for stroke prophylaxis, complicated by deep vein thrombosis (DVT) and acute limb ischemia (ALI). Despite treatment with fondaparinux, thrombocytopenia secondary to HIT persisted, and his arterial thrombosis progressed, leading to urgent angioplasty and thrombectomy. This clinical course raised concerns for autoimmune HIT (aHIT) refractory to fondaparinux or potential fondaparinux cross-reactivity. The patient was transitioned to argatroban postoperatively, resulting in rapid platelet recovery and clinical improvement. Our case highlights the limitations of fondaparinux in select HIT cases, particularly when aHIT is suspected, and underscores the need for vigilance in monitoring for treatment failure. This report adds to emerging data on alternative anticoagulation strategies for refractory HIT, including the potential role of direct thrombin inhibitors in severe cases.

## Introduction

Heparin-induced thrombocytopenia (HIT) is a rare, immune-mediated complication of heparin therapy that significantly increases the risk of life-threatening thrombotic events. HIT occurs in approximately 1-7% of patients exposed to heparin products and is characterized by a platelet drop exceeding 50%, typically within 5 to 10 days after heparin exposure [1]. This condition paradoxically creates a hypercoagulable state through platelet activation and aggregation, leading to complications such as venous thromboembolism (VTE), arterial thrombosis, and critical limb ischemia [2,3].

Fondaparinux, a synthetic pentasaccharide and indirect factor Xa inhibitor, has been used as an off-label alternative anticoagulant in the treatment of HIT, particularly in stable patients [4]. It does not bind to PF4 in the same way as unfractionated heparin (UFH) or low-molecular-weight heparin (LMWH), resulting in a markedly lower incidence of immune complex formation and subsequent platelet activation [5]. Although anti-PF4/heparin antibodies may develop during fondaparinux therapy, these antibodies typically have low reactivity toward PF4-fondaparinux complexes [6]. This pharmacologic profile underlies its favorable safety margin in HIT management. However, rare cases of fondaparinux-associated HIT have been reported, particularly in patients with prior sensitization or atypical immune responses [7-10].

In particular, autoimmune HIT (aHIT) represents a distinct and severe variant of HIT in which platelet-activating anti-PF4 antibodies can persist or arise without heparin exposure [11]. These antibodies activate platelets in the absence of heparin, resulting in delayed-onset thrombocytopenia, spontaneous venous thromboses, and potential resistance to standard therapies [12].

Acute limb ischemia (ALI), caused by sudden arterial obstruction with decreased limb perfusion, is a severe and potentially limb-threatening manifestation of HIT, though its association with HIT remains underexplored. This case report highlights a unique instance of worsening thrombosis and critical limb ischemia associated with the failure of fondaparinux therapy in HIT. Although fondaparinux is frequently used as an alternative anticoagulant in HIT, its ability to induce anti-PF4/heparin antibodies and rare cases of exacerbating thrombosis and failure of anticoagulation have been documented [2,7]. Our case highlights the limitations of fondaparinux in select HIT cases and underscores the need for vigilance in monitoring for treatment failure.

## Case presentation

A 69-year-old male with a history of stage IIIb small cell lung cancer, Chronic obstructive pulmonary disease (COPD), hypertension, and hypothyroidism presented to the emergency department with progressive shortness of breath, productive cough, and subjective fevers. On initial evaluation, he was found to have atrial fibrillation with a rapid ventricular response of unknown duration prior to presentation. Unfractionated heparin was initiated for stroke prophylaxis in the setting of new-onset atrial fibrillation with rapid ventricular response, for stroke prophylaxis. Also noted were leukocytosis (22.70 X 10*3/uL), hyponatremia (128 mmol/L), and a large right-sided pleural effusion.

**Figure 1:**
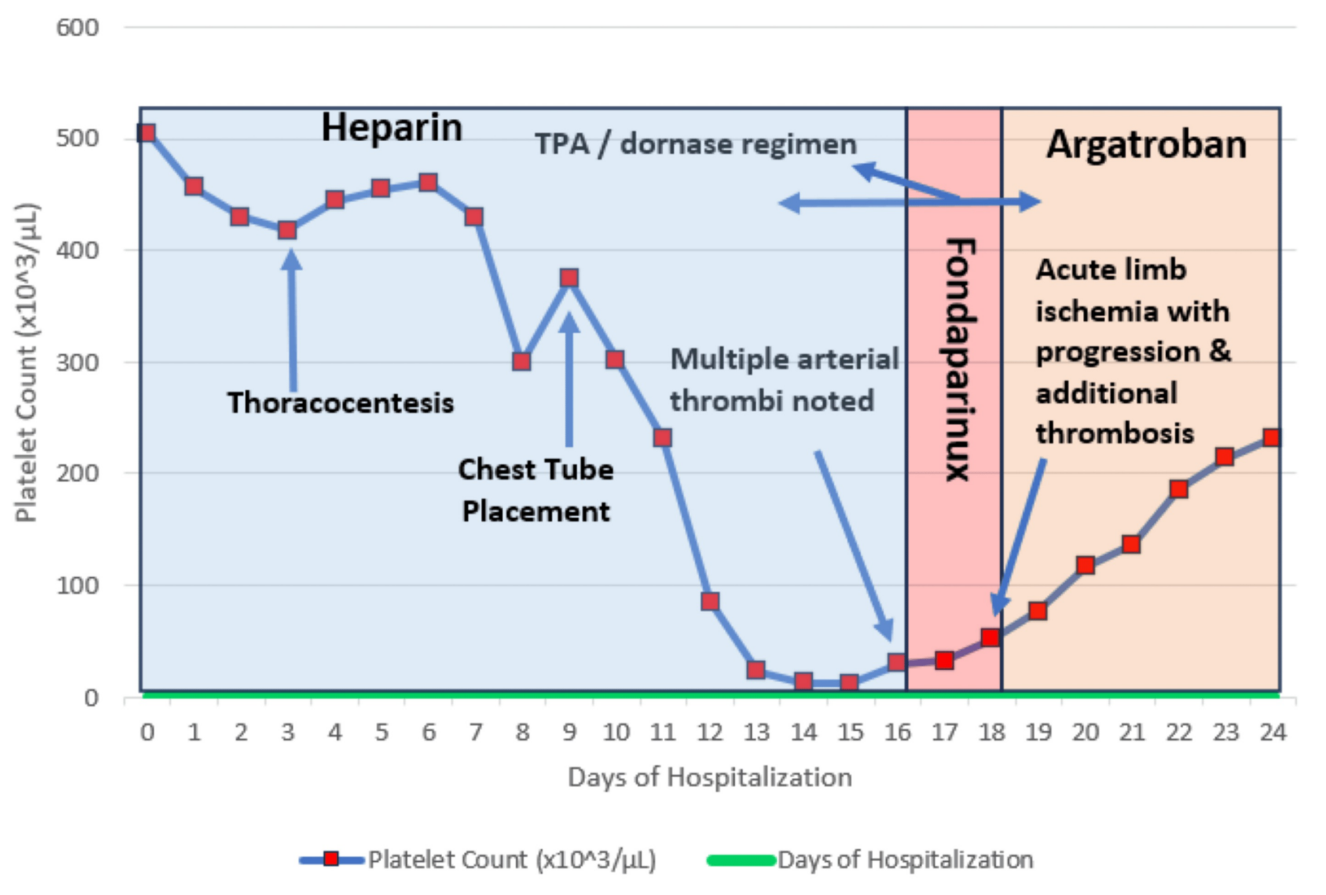
Daily platelet trend and key clinical events during hospitalization with treatment course overview The blue line with square markers depicts the patient’s platelet count (×10³/µL) plotted against hospital day. Background shading highlights the anticoagulant in use: light-blue (Days 1-15) = unfractionated heparin; rose vertical band (Days 16-18) = fondaparinux; light-orange (Day 18 onward) = argatroban. Annotated arrows identify major procedures (thoracentesis, chest-tube placement) and events (intrapleural TPA/dornase therapy, discovery of multiple arterial thrombi, and acute limb ischemia with additional thrombosis). The precipitous fall in platelets beginning on Day 10 culminated in a nadir of <10 ×10³/µL on Day 15, coinciding with radiologically confirmed arterial thromboses despite heparin cessation. Lack of platelet recovery and new thrombus formation while on fondaparinux prompted escalation to argatroban, after which platelet counts rose steadily with resolution of acute limb ischemia, illustrating fondaparinux failure and clinical response to direct thrombin inhibition. TPA: tissue plasminogen activator

His atrial fibrillation was managed with intravenous amiodarone, and unfractionated IV heparin dosed as a U/kg IV bolus then 18 U/kg/h with changes per nomogram per pharmacy without other exposures to anticoagulants was initiated for stroke prophylaxis on initial presentation (Figure 1). Thoracentesis revealed an exudative effusion, and a chest tube was placed for recurrent pleural effusion, which cultured Streptococcus constellatus. Empiric antibiotics and intrapleural tissue plasminogen activator (TPA)/dornase therapy were administered, leading to clinical improvement of the empyema.

**Figure 2:**
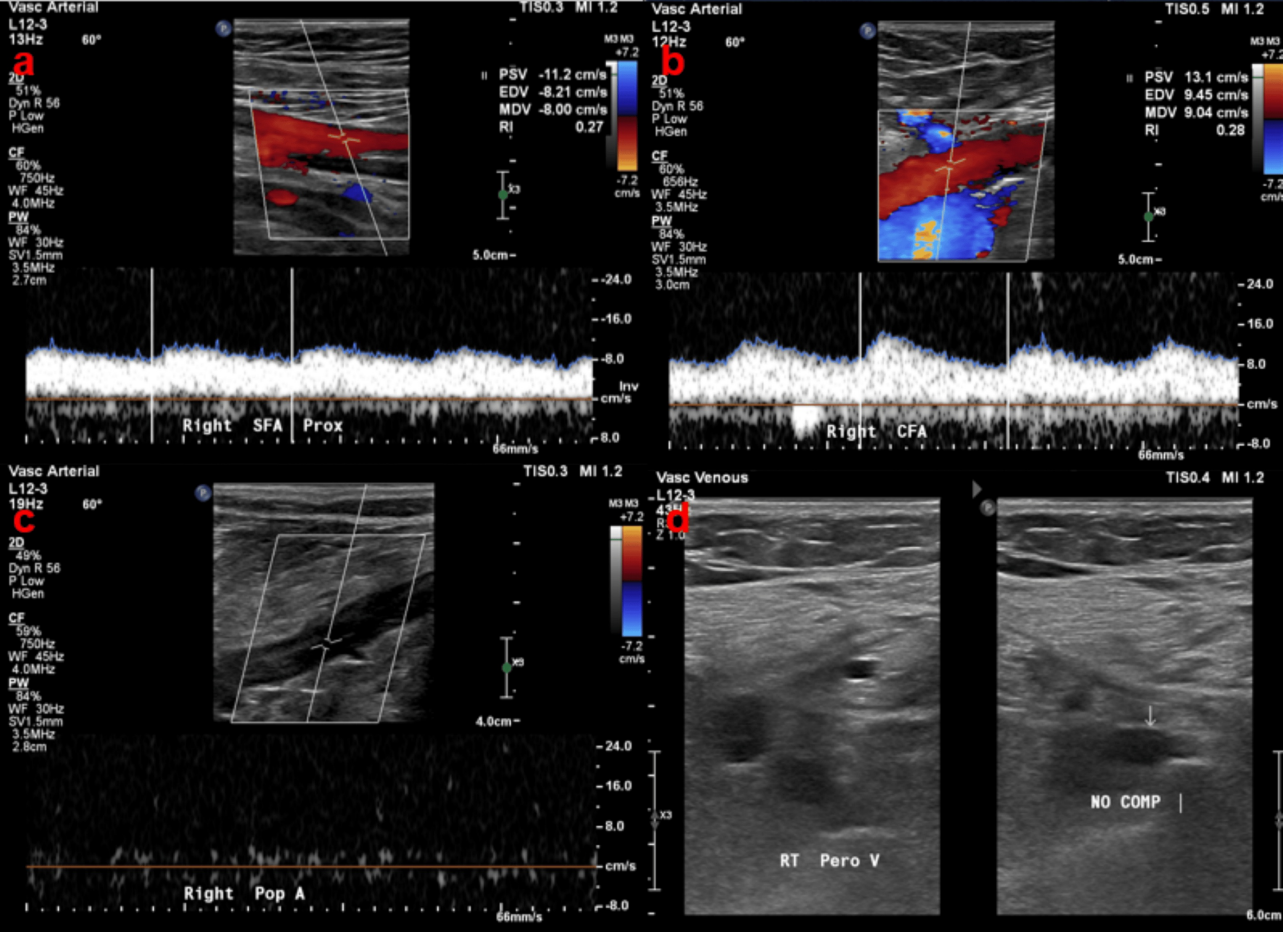
Duplex ultrasound of the right lower extremity demonstrating multilevel arterial inflow compromise and associated peroneal vein thrombosis a. Color + spectral Doppler of the right proximal superficial femoral artery shows a blunted, monophasic waveform, indicating markedly reduced forward flow with the indicator. b. Color + spectral Doppler of the right common femoral artery demonstrates a monophasic waveform, consistent with proximal (inflow) occlusive disease at the indicator. c. Spectral Doppler interrogation of the right popliteal artery reveals the absence of detectable flow, suggesting complete distal occlusion at the indicator. d. B-mode compression ultrasound of the right peroneal vein displays an intraluminal echogenic clot with failure to compress, confirming acute venous thrombosis at the indicator.

During the first week of admission (hospital days 1-7), the platelet count remained ≥ 420 × 10^9/L. Beginning on hospital day 8, it fell abruptly to 300 × 10^9/L and continued to decline, reaching a nadir of 5 × 10^9/L on hospital day 15 (Figure 1). Duplex ultrasonography performed on hospital day 16 revealed an acute right-peroneal deep-vein thrombosis (2d), and a same-day arterial duplex study showed total occlusion of the right tibioperoneal trunk, posterior-tibial, popliteal (2c) common femoral (2b), proximal superficial femoral (2a), and peroneal arteries (Figure 2). These findings prompted a full HIT workup. There was no sonographic evidence of arterial or venous thrombosis in the upper extremities in the subsequent study on the same day.

A 4T score was calculated, giving +1 for having a platelet count of a greater than 50% fall, with a nadir of less than 20. The initial platelet count fall was 8 days after the initiation of heparin, increasing the score by +2. The score was increased by +2 with noted arterial and venous thrombosis on day 16 of hospitalization after exposure to heparin. There were other possible causes of thrombocytopenia, including consumptive causes in the setting of chest tube placement, giving a +1 score. The 4T score on 7/15 was +6, with a high probability of HIT. Heparin was discontinued, and the patient was transitioned to Fondaparinux 7.5mg daily given subcutaneously. Laboratory testing was sent, later confirming the diagnosis with a positive anti-PF4 antibody (1.03) on day 16, and the serotonin release assay was positive on day 18 (SRA) (63% serotonin release at low-dose heparin).

**Figure 3:**
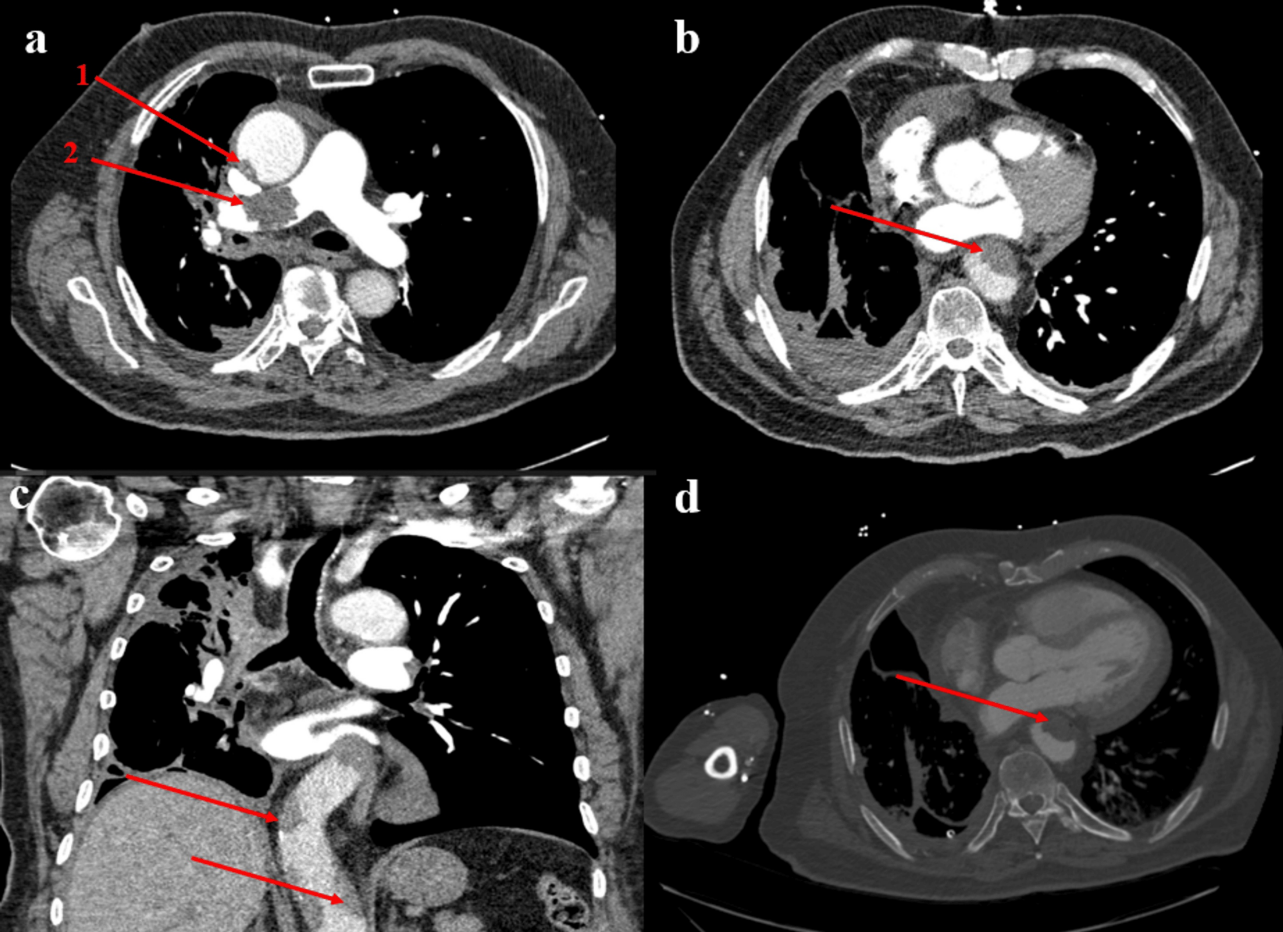
CTA imaging demonstrating arterial thrombus involving the thoracic aorta and pulmonary artery (a) Axial contrast-enhanced CTA image demonstrates a filling defect along the posterior wall of the ascending aorta (label 1), consistent with a non-occlusive mural thrombus. Additionally, there is a thrombus within the right main pulmonary artery (label 2), consistent with pulmonary embolism. These findings suggest concurrent arterial and pulmonary arterial thrombosis. (b) Axial CTA image shows a crescentic thrombus adherent to the anterior wall of the descending thoracic aorta, consistent with mural aortic thrombus (c) Coronal reformatted CTA image demonstrates a non-occlusive thrombus lining the descending thoracic aorta, visible along the right and left lateral walls. (d) Axial CTA in the soft tissue window reveals a large crescent-shaped thrombus within the descending thoracic aorta, consistent with mural thrombus formation more distally.

The patient’s clinical presentation progressed rapidly after two days of Fondaparinux administration. On day 18, the patient exhibited signs and symptoms of ALI in the right lower extremity while the patient was still receiving fondaparinux, including sudden right leg pain, inability to walk, a cold right lower extremity, and an absence of pulses in the affected limb. On rapid CTA chest imaging, significant pulmonary emboli and aortic thrombus were noted (Figure 3).

**Figure 4:**
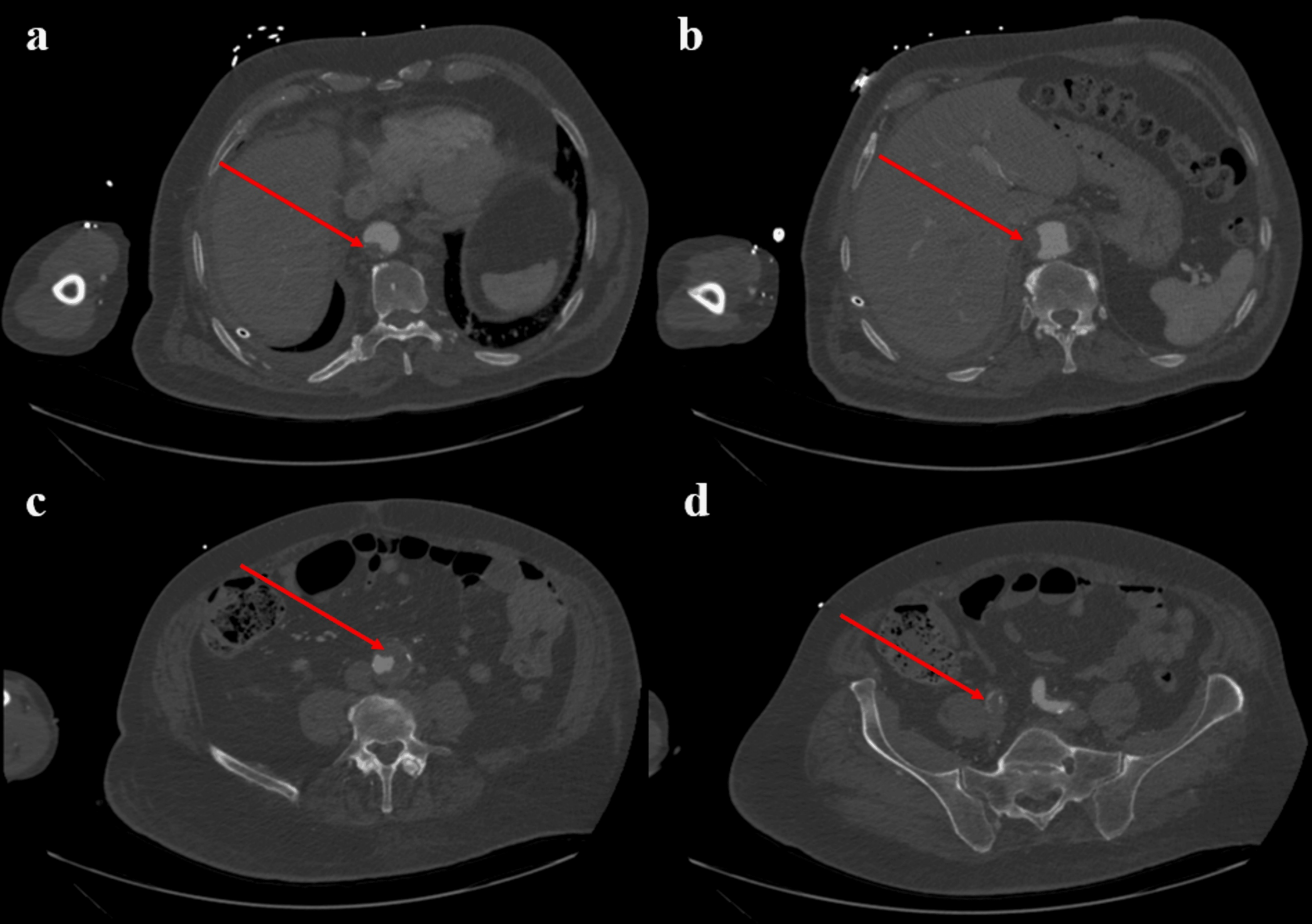
CT angiography of lower extremities with runoff imaging aorta and common iliac arteries (a) Axial CTA image at the level of the upper abdominal aorta just below the diaphragm shows a mural thrombus along the posterior wall; (b) Axial CTA at the level of the renal arteries demonstrates eccentric thrombus along the lateral portions of the abdominal aortic wall; (c) Axial CTA at the mid-abdomen reveals persistent crescentic mural thrombus within the distal abdominal aorta, tracking inferiorly; (d) Axial CTAa at the pelvic inlet shows thrombus extending into the right common iliac artery.

In addition, rapid CTA of the abdomen was performed on day 18, noting extensive mural thrombus throughout the visible aorta, including significant involvement of the aneurysmal infrarenal abdominal aorta (Figure 4).

In addition, an occlusive or subtotal occlusive thrombus in the distal right common iliac artery extending into the right external iliac artery, with narrowing and eventual occlusion of the distal right superficial femoral artery, and occlusion of the right popliteal artery was noted (Figure 4). On the left side, there is abrupt occlusion or subtotal occlusion of the distal left superficial femoral artery, with reconstitution of the left popliteal artery and patency of the proximal left calf arteries.

**Figure 5:**
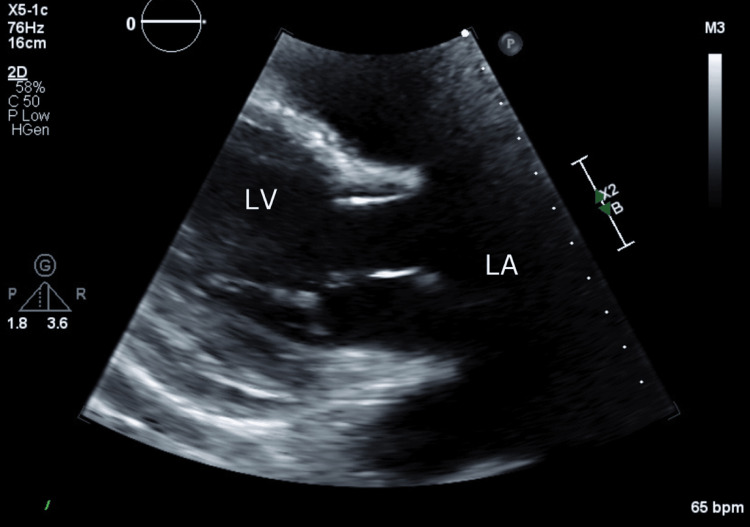
Parasternal long-axis view on transthoracic echocardiogram demonstrating the absence of vegetation and thrombus on the mitral valve as a source of embolization No intracardiac thrombus in the left atrium (LA), left ventricle (LV), or mitral valve during evaluation for a potential cardioembolic source.

On day 18 of hospitalization, a transthoracic echocardiogram (TTE) was obtained to evaluate for a cardiac source of embolism in the setting of new arterial thrombosis (Figure 5). The study revealed no evidence of intracardiac thrombus, left atrial enlargement, or valvular pathology, and left ventricular systolic function was preserved. As there was no thrombus, atrial fibrillation with subsequent cardiac embolus was a less likely source of the observed arterial thrombi.

With confirmed ALI, urgent angioplasty was done with stenting of the right iliac artery and thrombectomy of the right superficial femoral and popliteal arteries. The patient was transferred to the ICU for closer monitoring.

Due to persistent thrombocytopenia and concerns about hypercoagulability potentially linked to fondaparinux, he was transitioned to argatroban 2 µg/kg/min IV titrated to activated partial thromboplastin time (aPTT) on day 18 after the surgery (Figure 1). The patient tolerated the surgery and argatroban well and had a significant improvement in ALI symptoms within a few days. The patient could be transferred from the ICU within three days of intervention and treatment with argatroban. Platelet recovery was noted by day 24 of hospitalization, allowing for a transition to long-term anticoagulation with apixaban. He was discharged on day 24 with a prescription for apixaban for anticoagulation. Follow-up was planned to monitor platelet counts, anticoagulation, and for surveillance of thrombotic events. On follow-up, the patient was noted to have a progression of lung cancer, now metastasized to the liver, two months later.

## Discussion

Heparin-induced thrombocytopenia: overview and pathogenesis

HIT is a rare but potentially life-threatening complication, affecting 0.1% to 5% of patients receiving heparin [1]. It paradoxically presents as a hypercoagulable state, with thromboembolic complications, such as venous thromboembolism (VTE), arterial thrombosis, and thrombotic stroke, occurring in 30% to 50% of cases. The pathogenesis of type II HIT involves the formation of immunogenic PF4-heparin complexes, which are targeted by IgG antibodies that activate platelets via FcγRIIa. This activation triggers a prothrombotic cascade involving microparticle release, thrombin generation, and platelet consumption within fibrin thrombi, leading to sustained thrombocytopenia and systemic hypercoagulability [5,6,11]. The risk of HIT is lower with low-molecular-weight heparin (LMWH) compared to unfractionated heparin (UFH), due to reduced PF4-heparin complex formation and antibody development [12].

Fondaparinux, Autoimmune HIT, and Delayed-Onset HIT

Fondaparinux, a synthetic pentasaccharide anticoagulant that selectively inhibits factor Xa, offers benefits such as convenient once-daily subcutaneous dosing, an extended half-life, and minimal need for monitoring [13]. Fondaparinux paradoxically serves as both a potential cause and a widely used treatment for HIT. It has been associated with autoimmune HIT (aHIT) or delayed-onset HIT, a severe subtype of HIT characterized by heparin-independent anti-PF4 antibodies that activate platelets even in the absence of heparin. These antibodies, shown in assays to cause strong serotonin release, have been linked to severe complications such as bilateral adrenal necrosis, deep vein thrombosis (DVT), pulmonary embolism, and arterial thrombosis [7-9].

Our case is particularly noteworthy, as the patient developed arterial thrombosis before receiving fondaparinux, yet progressed to ALI with increased arterial thrombosis after two days of treatment. In addition, the patient’s platelet count did not appreciably improve after two days of therapy, with a sharp clinical and laboratory improvement observed after initiating argatroban (Figure 1). We did not assess fondaparinux cross-reactivity with HIT antibodies or the persistence of serotonin release assay (SRA) positivity in this case, making its role in treatment failure speculative. However, the patient's persistent thrombocytopenia, worsening arterial and venous thrombosis, and rapid platelet recovery after switching to argatroban suggest a possible connection.

While the patient had documented heparin exposure and tested positive for HIT via both PF4 and serotonin release assay (SRA), the continued progression of thrombosis and thrombocytopenia after heparin discontinuation raised concern for autoimmune HIT (aHIT). aHIT is characterized by heparin-independent platelet activation that persists even after stopping heparin. However, it is also possible that fondaparinux may have contributed to ongoing platelet activation through rare cross-reactivity. Therefore, we interpret this case as consistent with either aHIT or the delayed-onset HIT form of heparin that was refractory to Fondaparinux treatment, marked by ongoing thrombotic activity despite cessation of heparin.

Clinical Presentation of HIT, Diagnosis, and Management

HIT often manifests as thrombocytopenia, defined by a platelet count below 150 × 10⁹/L or a 30-50% decline from baseline, typically occurring 5-14 days after heparin exposure. Thrombosis can sometimes develop before a significant decline in platelet count, and the severity of thrombocytopenia correlates with a higher risk of thrombotic complications [2,3]. Clinical tools, such as the 4Ts and HEP scores, help assess pre-test probability, guiding further evaluation with PF4 ELISA testing and confirmatory serotonin release assays (SRA). The 4T score is based on four components: the degree of thrombocytopenia, the timing of platelet count decline relative to heparin exposure, the presence of thrombosis or other sequelae, and the likelihood of other causes of thrombocytopenia. Each category is scored from 0 to 2, yielding a total score between 0 and 8. A score of 0-3 suggests low probability, 4-5 intermediate probability, and 6-8 high probability of HIT, and guides decisions on further SRA with intermediate or high scores.

In cases of HIT, heparin or the offending agent should be discontinued immediately, and an alternative anticoagulant, such as argatroban, bivalirudin, fondaparinux, or danaparoid, should be initiated without delay. Recent studies have also explored the use of direct oral anticoagulants (DOACs). Anticoagulant choice depends on factors such as renal and hepatic function, procedural requirements, medication cost, and patient acuity [13]. Current guidelines suggest fondaparinux for stable patients with moderate bleeding risk, whereas argatroban or bivalirudin is preferred in unstable patients due to their shorter half-lives [13].

Our case highlights a potential risk of disease progression or treatment failure with fondaparinux in a less stable patient with HIT, emphasizing the need for careful patient selection.

After examining the literature for similar cases of fondaparinux failure, several reports document instances in which fondaparinux treatment failure led to worsening thrombosis (Table 1) [10,14,15]. Reports of fondaparinux-associated HIT have shown positive serotonin release assays (SRA), indicating potential antibody cross-reactivity and direct platelet activation [10,14,20]. Similar to our case, fondaparinux failure has been linked to pro-inflammatory conditions, such as malignancy, infection, and recent surgery, which may exacerbate the hypercoagulable state [9,10,14,15].

**Table 1:** Summary of reported cases of fondaparinux failure in heparin-induced thrombocytopenia: patient characteristics, HIT characteristics, thrombotic complications, and management outcomes HIT: heparin-induced thrombocytopenia; LMWH: low-molecular-weight heparin; DVT: deep vein thrombosis; NSTEMI: non-ST-elevation myocardial infarction; UFH: unfractionated heparin; DIC: disseminated intravascular coagulation; IVIG: intravenous immunoglobulin; IVC: inferior vena cava

Author (Year) and Citation	Patient Characteristics	Fondaparinux Indication	Prior Heparin Exposure	Nadir Platelet Count	Timing of Nadir (Days)	Thrombotic Complications	4T Score	Serotonin Assay Results	Anti-PF4 Testing	Other Clinical Events Associated with HIT	Management	Outcome
Rota et al. [8]	74-year-old female; history of HIT with LMWH, post-total right hip replacement	Thromboprophylaxis	Yes, remote LMWH-induced HIT	50× 10⁹/L	11	None reported	Not reported	Not reported	Strongly positive	Following total hip replacement	Discontinued fondaparinux	Recovery
Salem et al. [9]	67-year-old male; post-knee arthroplasty	Thromboprophylaxis	None	35 × 10⁹/L	10	Arterial thrombotic stroke, bilateral DVT	Not reported	Not reported	Strongly positive 2.803 units	Persistent vegetative state	Discontinued fondaparinux; initiated argatroban, followed by warfarin	Persistent vegetative state
Warkentin et al. [7]	74-year-old female; interstitial pulmonary fibrosis, with urosepsis	Thromboprophylaxis	None	51 × 10⁹/L	10	None reported	6	Positive	Strongly Positive, 3.00	NSTEMI, mechanical ventilation	Discontinued fondaparinux; initiated argatroban	Recovery
Manji et al. [14]	70-year-old female; post-glioblastoma resection	Thromboprophylaxis following HIT from heparin thromboprophylaxis	Yes, UFH thromboprophylaxis	25 × 10⁹/L	12	Symptomatic catheter-associated upper-extremity DVT, DIC, lower-limb DVT	Not reported	Positive with UFH and fondaparinux	Positive	Persistent thrombocytopenia, DIC, and glioma resection	High-dose IVIG and rivaroxaban; fondaparinux discontinued	Recovery
Modi et al. [15]	68-year-old male; metastatic prostate cancer	Thromboprophylaxis for a history of HIT	Yes, prior HIT (1 year earlier)	75 × 10⁹/L	14	Pulmonary embolism, femoral vein thrombosis	6-7	Not reported	Positive	Acute respiratory failure	Discontinued fondaparinux; initiated argatroban	Fatal
Malik et al. [16]	46-year-old male; diagnosed with NSTEMI	Thromboprophylaxis and NSTEMI	None	90 × 10⁹/L	2	None reported	Not reported	Not reported	Not reported	None reported	Discontinued fondaparinux; initiated lepirudin	Recovery
Burch et al. [17]	47-year-old male; post-total knee replacement	Thromboprophylaxis	Yes prior to nadir UFH	50 × 10⁹/L	19	Inferior vena cava thrombus, bilateral adrenal hemorrhage	Not reported	Not reported	Positive, 2.807	Abdominal distention, fever	Discontinued fondaparinux; initiated lepirudin	Recovery
Burch et al. [17]	63-year-old male; post-total knee replacement	Thromboprophylaxis following knee replacement	None	38 × 10⁹/L	10	Right lower extremity ischemia, splenic and renal infarcts	Not reported	Positive	Positive 3.08	Nausea, vomiting, abdominal pain	Discontinued fondaparinux; initiated argatroban	Recovery
Maurer et al. [18]	11-year-old female; history of lower extremity DVT and pulmonary embolism	HIT-associated thrombosis	Yes, heparin-induced thrombocytopenia (HIT)	53 × 10⁹/L	49	Recurrent DVT, IVC occlusion, pulmonary embolism	7	Not reported	Positive	Re-thrombosis after warfarin and fondaparinux use	Discontinued fondaparinux; initiated argatroban	Recovery
Sartori et al. [19]	86-year-old male; post-carotid endarterectomy, autoimmune HIT, CKD	Thromboprophylaxis	Yes, a single intraoperative UFH dose	43 × 10⁹/L	27	Isolated distal DVT (calf veins)	5 (2.35)	Not reported	Positive	Autoimmune HIT with delayed onset, persistent thrombocytopenia	Discontinued fondaparinux; initiated rivaroxaban	Recovery (no recurrence after 3 months)

Fondaparinux failure after classic, heparin-triggered HIT - the sequence we observed - has been documented in Manji et al., Burch et al., Maurer et al., and Sartori et al. [14,17,18,19]. Conversely, fondaparinux-induced HIT is reported by Rota et al., Salem et al., Warkentin et al. (2007 and 2012), Modi et al., Malik et al., and Burch et al., whereas Sartori et al. explicitly classed their episode as autoimmune HIT, defined by heparin-independent, platelet-activating anti-PF4 antibodies [8-10,15-17,19]. This side-by-side presentation purposefully spans both scenarios as fondaparinux, whether involved in the failure of treatment of HIT or causing HIT, is a helpful repository of cases to demonstrate the clinical circumstances in each scenario. Although our case is consistent with autoimmune HIT, the absence of confirmatory testing for fondaparinux-dependent platelet activation (e.g., modified SRA or PF4-enhanced assays) limits definitive classification. Therefore, a diagnosis of either refractory HIT or aHIT remains clinically plausible but unconfirmed. 

Multiple treatments have demonstrated efficacy following fondaparinux failure, including argatroban and lepirudin [8,10,14-19]. Further investigation is warranted regarding the addition of intravenous immunoglobulin (IVIG) in cases of suspected or confirmed aHIT associated with fondaparinux. Our case aligns with prior reports suggesting that aHIT may present with a significantly lower platelet nadir, whereas other documented cases of fondaparinux failure typically involved platelet counts above 20 × 10⁹/L (Table 1) [2].

Similar to our patient who developed ALI, several patients experienced severe complications, such as arterial thrombotic stroke, pulmonary embolism, and ALI, reinforcing that fondaparinux failure is often associated with both venous and arterial thrombosis rather than isolated venous thromboembolism (VTE) [9,14,15]. This is in alignment with either a severe delayed HIT presentation or aHIT.

## Conclusions

The management of HIT, especially in cases of fondaparinux failure in probable aHIT, highlights the complexities of balancing therapeutic benefits against rare but severe complications. Fondaparinux, despite its utility, may not be an ideal anticoagulant in these cases. We present a rare case of HIT with associated ALI, which is an uncommon presentation. HIT remains a serious clinical concern, requiring prompt diagnosis, careful anticoagulant selection, and continued research to enhance treatment outcomes in complex cases. Our case underscores the importance of recognizing early warning signs of treatment failure in HIT and considering alternative anticoagulants when necessary. Given the potential for autoimmune mechanisms in refractory cases, further investigation is needed to determine optimal therapeutic approaches for aHIT. Future research should also explore the role of immunomodulatory therapies, such as intravenous immunoglobulin (IVIG), in cases of fondaparinux failure, to mitigate the hypercoagulable state associated with aHIT.
